# A single center 9-year experience in IVC filter retrieval - the importance of an IVC filter registry

**DOI:** 10.1186/s42155-022-00291-5

**Published:** 2022-03-05

**Authors:** Mark Sheehan, Kristopher Coppin, Cormac O’Brien, Andrew McGrath, Mark Given, Aoife Keeling, Michael J Lee

**Affiliations:** 1 Royal College of Surgeons in Ireland, Dublin, Ireland; 2 Department of Radiology, Beaumont Hospital, Dublin, Ireland

## Abstract

**Background:**

To evaluate Inferior vena cava (IVC) filter retrieval practices over a 9-year period at an academic hospital with a prospectively maintained IVC filter registry.

**Method:**

An IVC filter registry was maintained prospectively within our institution. We reviewed cases between August 2011 and June 2020, following filter status, retrieval plans, and eventual retrieval date. The validity of the database was cross referenced with a Picture Archiving and Communication System and patient records.

**Results:**

Three hundred forty-three patients had IVC filters inserted. Three filter types were used, Celect (Cook Medical) in 189, Gunther Tulip (GT) (Cook Medical) in 65, ALN (ALN) in 89. 196 (57%) filters were retrieved, 108 (31.5%) were made permanent, 36 (10.5%) died before retrieval, and 3 (1%) were yet to be retrieved.

Retrieval rates were 92.5% overall (86% for GT, 93% for Celect and 94.5% for ALN). The mean dwell time for successful retrieval was 59 days with the majority of insertions (85%) removed in under 100 days. Failed initial retrieval occurred in 23 patients, 10 (43%) were retrieved at second attempt, 13/23 filters remained in-situ and were deemed permanent.

**Conclusion:**

The removal of IVC filters, when indication for insertion has past, is no longer the sole responsibility of the referring physician but also the responsibility of the Interventionalist. Our retrieval rates of 92.5% of eligible IVC filters highlights the value of maintaining a prospective IVC filter registry.

## Introduction

Venous thromboembolism (VTE) is a collective term for deep vein thrombosis (DVT) and pulmonary embolism (PE); these are common conditions associated with significant rates of patient morbidity and mortality. The overall VTE estimated average annual incidence rate among persons of European ancestry ranges from 104 to 183 per 100,000 person-years.[1].

The standard care given for VTE is oral anticoagulation therapy, however in a subset of cases oral anticoagulation fails to prevent VTE or anticoagulation is contraindicated; at this point clinicians may find the insertion of an inferior vena cava (IVC) filter as the most suitable option for the treatment of VTE. Greenfield established the IVC filter in 1973 as means of preventing PE by intercepting potential emboli originating in or distal to the IVC. The introduction of retrievable filters in 2003 expanded the utility of IVC filters in patients with transient indications and has coincided with increased IVC filter insertion.[2].

IVC filters which are not retrieved can pose a risk to patients because of their association with complications such as vena cava wall perforation, thrombosis or stenosis, as well as filter fracture or migration.[3–7] In light of the potential risk non-retrieved filters pose against the general population; the U.S. Food and Drug Administration (FDA) released a safety notice in 2010, which alerted Interventionalists implanting these medical devices as to their shared responsibility with physicians in following up with each individual patient to retrieve these devices as soon as deemed clinically appropriate.[8] This prompted the establishment of an IVC registry in our institute, to prospectively record and track retrievable IVC filters that were inserted.

The FDA released a subsequent notice in May of 2014 with a mathematical model which suggested that once PE risk has resolved, the risk/benefit profile starts to favour the removal of the IVC filter within 29 to 54 days of insertion.[9] Angel et al. produced a systematic review in 2011, including 37 studies and over 6800 patients, on IVC filter practices and quoted a mean retrieval rate of 34%, and mean dwell times of 72 days.[6] More recently CIRSE (Cardiovascular and Interventional Radiology Society of Europe) and BSIR (British Society of Interventional Radiology) have shown improved filter rates when they set up online registries for institutes with retrieval rates quoted of up to 92%.[10, 11] However, there is evidence to support that with dedicated follow-up retrieval rates approach 60%.[4].

The aim of this study is to review our retrieval practices over a 9-year period to determine the retrieval rates for IVC filters using a prospectively managed IVC filer database with secondary outcome measures including dwell times, insertion indications, and reasons of permanent filter insertions.

## Methods

An IVC filter registry was prospectively collated within our institution. The responsibility of database maintenance is shared between a nominated Interventional Radiology (IR) fellow and departmental IR administration staff. Details of the database were stored on an Microsoft Excel Spreadsheet.

We reviewed the database from inception in August 2011 until June 2020. We assessed the registry against a Picture Archiving and Communication System and electronic patient records to ensure data entry was accurate. Data entry consisted of IVC filter status, dwell time, retrieval date, retrieval complications, insertion indications, access route used, malignancy diagnosis, indications for permanent filters, failed attempts and extra manoeuvres performed for retrieval.

Before filter insertion, patients were streamed into 3 different patient groups; (1) those filters placed with an ‘intent to retrieve’, (2) filters deemed to be ‘permanent’ on insertion and (3) the ‘unspecified’ group - the decision whether the filter was permanent or to be retrieved was not possible to make at the time of insertion. The status of the filter was reviewed regularly with the referring physician. A final filter status for the ‘unspecified’ was made within 3 months and recorded when a final decision was made after consultation with the referring physician.

All patients who had an IVC filter inserted at our institution were recorded in the registry and thus were eligible for inclusion. A small number of patients had IVC filters retrieved at our institution but their filters were inserted elsewhere and thus were excluded as there was insufficient data available.

**Figure 1:**
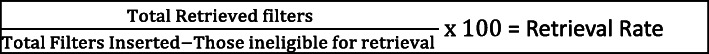
Fig. 1

IVC filter retrieval rates were calculated as follows: total retrieved filters/ (total filters inserted – ineligible for retrieval) x 100 (Fig. 1). Ineligibility for retrieval occurred if a filter was decided to be left permanently (excluding a failed attempted retrieval - who were deemed eligible) and those cases who died prior to retrieval attempt were also considered ineligible. Patients with a filter yet to be retrieved i.e. loss to follow up, were considered eligible and are included in the retrieval rate calculation. This method of calculation has been documented in previous studies.[4].

## Results

There were 343 patients entered into the database (174 male and 169 female) over the 9-year period. The mean age for men was 59.8 years (range 19–87) and for women was 58.8 years (range 18–88). All IVC filter insertions were performed in our institution, 7 filters were retrieved in an external institute, the remaining filters were retrieved in our hospital.

The IVC filter types inserted were the Celect filter (Cook Medical, Bloomington, Indiana) in 189, the Gunther Tulip (GT) (Cook Medical, Bloomington, Indiana) in 65, ALN (ALN, Ghisonaccia, France) in 89.

The access routes for insertion approach were the right internal jugular vein in 278 cases (81%), right common femoral vein in 58 cases (17%) and the left common femoral vein in 7 cases (2%). A diagnosis of malignancy at the time of filter insertion was reported in 115/343 (33.5%) patients. 30 of these 115 patients (26%) had a permanent IVC filter inserted de novo. Limited life expectancy independently or combined (Table 1) constituted 53 (49%) permanent filters with 9/53 (17%) also having a malignancy status.

**Table 1:** Indications for Permanent Filters (*n* = 108)

Indications for Permanent Filters	Number of Cases
Limited Life Expectancy	30 (27.7%)
Long-term CI (Contraindication) to AC (Anti-Coagulation)	28 (25.9%)
Failed Retrieval	13 (12%)
VTE (Venous Thromboembolisim) on therapeutic AC	10 (9.3%)
Patient Preference	4 (3.8%)
Multiple - Limited Life Expectancy + Long-term CI to AC 13 (12.6%) - Limited Life Expectancy + VTE on AC 10 (9.7%)	23 (21.3%)
Overall total	108

Indications for insertion of IVC filters were separated into 6 categories: contraindication to anticoagulation in setting of VTE in 147 (43%), pre-operative prophylaxis (Patients with a previous history of VTE undergoing major surgery) in 117 (34%), pharmaco-mechanical thrombectomy in 47 patients for iliofemoral DVT (14%), VTE on therapeutic anticoagulation in 20 (6%), extensive VTE in 5 (1%) and multiple indications in 7 (2%), as shown in (Table 2).

**Table 2:** Indications for Filter Insertion (*n* = 343)

Indications for Filter Insertion	Number of Cases (%)
CI (Contraindication) to AC (Anti-Coagulation)	147 (42.7%)
Pre-operative prophylaxis	117 (34.1%)
Pharmaco-mechanical thrombectomy (PMT)	47 (13.7%)
VTE (Venous Thromboembolisim) on AC	20 (5.8%)
Extensive VTE	5 (1.7%)
Multiple - CI to AC + extensive VTE 2 (0.6%) - CI to AC + PMT. 2 (0.6%) - Pre-operative + VTE on AC 1 (0.3%) - PMT + extensive VTE 1 (0.3%) - pre-operative + CI to AC 1 (0.3%)	7 (2.0%)
Overall total	343

**Figure 2:**
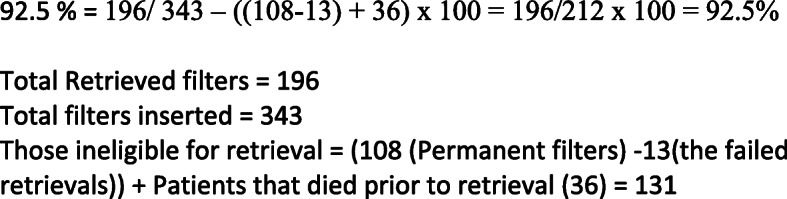
Fig. 2

Of the 343 filters inserted, 196 (57.1%) filters were retrieved, 108 (31.5%) filters were deemed permanent, 36 (10.5%) patients died during follow-up, and 3 (0.9%) patients were yet to be retrieved or lost to follow up. The overall retrieval rate was 92.5% (Fig. 2), with a retrieval rate of 86% for GT, 93% for Celect and 94.5% for ALN (Table 3).

**Table 3:** Retrieval Rate by Filter Type (*n* = 343)

Filter Type	Filter Status				Total	Retrieval	
	Retrieved	Permanent	In situ	Deceased		Failed *	Successful retrieval
Gunther Tulip	18	42	0	5	65	3	86%
Celect	126	44	2	17	189	8	93%
ALN	52	22	1	14	89	2	94.5%
Overall total	196	108	3	36	343	13	92.5%

* Failed category totals are a subset of the permanent category totals

The mean dwell time for successful retrieval was 59 days (median 42, range 2-390 days) with the majority of insertions (85%) removed in under 100 days (Table 4).

**Table 4:** Mean dwell time by Filter Type (*n* = 209)

Filter Type	Successful cases		Failed Cases		Length of Time until retrieval	
	Quantity	Mean No. of days until retrieval	Quantity	Mean No. of days until attempted retrieval	Max days	Min days
Gunther Tulip	18	50	3	63	144	15
Celect	126	58	8	73	336	2
ALN	52	67	2	81	390	4
Total	196	59	13	72		

Before insertion, 61 patients were streamed into the ‘permanent filter’ group, 48 were streamed into the ‘unspecified plan’ group and the remaining 234 were streamed into the ‘intent to retrieve’ group.

Of the 48 patients in the ‘unspecified plan’ group a later decision with regard to the filter being retrieved or deemed permanent was made within 3 months of filter insertion after discussion of the clinical condition of the patient with the referring physician. Of these 48 patients, filters were assigned permanent status in 35 due to deteriorating clinical illness and poor life expectancy and 13 were assigned to the intent to retrieve group. Of the latter 13 patients, 6 filters were retrieved ,6 died before IVC retrieval and 1 was lost to follow up.

In the ‘intent to retrieve’ group, 190 (81.2%) of 234 filters were retrieved either on first or second attempt. 30 (12.8%) patients of the 234 died prior to retrieval, 12 (5.1%) were made permanent and 2 (0.9%) were lost to follow up.

As stated, 61 filters were deemed permanent from the time of insertion, all 61 remained in situ. On final review, filters were assigned the status of permanent in 108 (31.5%), (42 Gunther Tulip, 44 Celect, and 22 ALN). The clinical circumstances under which the determination for the filter to remain permanently is shown in Table 1. This means that a permanent status was applied to a filter in 47 patients, who before insertion were streamed initially into the ‘intent to retrieve’ group or ‘unspecified plan’ group.

Unsuccessful retrieval at the first attempt occurred in 23 patients. Table 5 shows the causes for failed retrievals on the first attempt. 13 (57%) patients who had an unsuccessful retrieval on the first attempt went on to become permanent after discussion with the patient and referring teams (these cases were then considered a ‘failed retrieval’) and 10 (43%) were successfully retrieved on the second attempt. Advanced manoeuvres were used in 19 cases overall with 13 (68%) resulting in successful retrieval. 9 (39%) of the 23 initial failed attempts had advance manoeuvres performed. In filters with thrombus at the time of retrieval, the patients remained on anticoagulation and a further date for retrieval planned in 2–3 weeks time. Retrieval on second attempt was successful in 50% (5/10) of these cases.

**Table 5:** Reasons for failed initial retrieval (*n* = 23)

Reason for failed initial retrieval	Number of Cases
Thrombus in filter	10 (43.5%)
Endothelialized	5 (21.7%)
Re-inserted above filling defect	2 (8.7%)
Filter tilted/ Unable to snare	1 (4.4%)
Access route thrombosed	1 (4.4%)
Multiple - Endothelialized and thrombus in filter 3 (13.0%) - Endothelialized and unable to snare 1 (4.4%)	4 (17.4%)
Overall total	23

The vast majority of retrievals was performed using local anaesthetic only, typically 10mls of 1% lidocaine. In some cases, light conscious sedation was used, a combination of Fentanyl and Midazolam were administered. Retrievals performed under general anaesthetic usually occurred when the patient was already intubated in an intensive care setting. The ALN extraction device (ALN, Ghisonaccia, France) was the preferred method of retrieval, although a range of snare devices were also used routinely depending on operators preference.

The most commonly employed advanced manoeuvre was the loop snare technique, this involves engaging a reverse curve catheter in the struts of the filter and inserting a hydrophilic wire cranially. The wire is then snared and externalized, thus used as a counter force as the co-axial sheath is passed over the collapsed IVC filter. Another advanced manoeuvre performed in two cases involved passing a wire between the endothelialized hook and wall of the IVC. A balloon venoplasty was performed in an attempt to separate the hook from the IVC wall and thus allow for the hook to be snared. A semi rigid forceps with a larger 16 French sheath was used successfully in 3 patients when some of the above retrieval methods failed.

## Discussion

It is the shared responsibility of the referring physician and the interventional radiologist to ensure that IVC filters are removed at the earliest juncture once the indication for placement has resolved. While the use of IVC filters reduces the risk of VTE in the short-term it increases the risk of DVT in the long term [4, 9].

The use of IVC filter databases or registries have become common place within the IR community [10, 11] since recommendations from the FDA was published in 2010 advising the removal of retrievable IVC filters as soon as possible.[8] Different methods of improving retrieval rates have been investigated, examples include prospective registries, letters to referring physicians, scheduling IVC retrieval dates at the time of insertion and even IVC filter clinics. In our institute it was the responsibility of the IR who inserted the filter to document in the registry the planned retrieval date and schedule the patient for same. Non-specified retrieval plans were documented in 14% (*n* = 48) of the total cases, in the cases lost to follow up 33% (*n* = 1/3) had a non-specific retrieval plan documented. This in itself highlights the importance of having a retrieval date in place as soon as the IVC filter has been inserted. Sutphin et al. compared three patient cohorts, firstly a baseline group with a retrieval rate of 8%, secondly a “letters” cohort with a retrieval rate of 40% (the referring physician is contacted by letter) and finally a third “Prospective” cohort with a retrieval rate of 52% (these patients were scheduled for a clinic appointment at the time of the IVC insertion).[4] Similarly Minocha et al. demonstrated an increase of 29–60% after an IVC clinic and prospective database were implemented.[12].

There is no definitive nomenclature documented in the literature for the calculation of the retrieval rate and care needs to be taken when comparing retrieval rates. We have reported a retrieval rate of 92.5%, which means we removed 92.5% of the IVC filters that were eligible for retrieval, the formula of which can be seen in Fig. 1. This formula for calculation was also used by Sutphin et al.[4] Minocha et al. reported a retrieval rate of 29% in pre-clinic years to 60% in post-clinic years for optional/temporary filters, however it does not include the 33 patients who had filters made “permanent” after insertion and therefore would have a retrieval rate of 91% using our calculations.[12] In 2015 CIRSE produced an online registry for multiple international centers and published a similar retrieval rate of 92% for eligible retrievable filters.[10] Similar high retrieval rates have been reported by De Gregorio et al. (SERVEI- REFiVeC registry) removing 76.9% of all retrievable IVC filters but when augmented for deaths or change of status to permanent the adjusted retrieval rate is of 94.15%.[13] De Gregorio et al. highlights well the heterogenous nature of comparing rates between papers due to different study designs.

However these high retrieval rates are not seen across the board, Sarostek et al. reported a retrieval rate of 8.5% on 679 retrievable IVC filters in a large single center institute.[14] As discussed with the introduction of IVC clinics, letters to physicians and registries has greatly improved these figures. [4, 10–12]

108 filters were made permanent, 13 of these were due to failed retrieval (these were included in the calculation as failed retrievals) In 7 of these 13 failed retrievals, no advanced manoeuvres were attempted. The mean dwell time for these failed retrievals were 63 days for GT filters, 73 for Celect and 81 for ALN filters. At greater dwell times advanced maneuvers become essential to ensure high successful retrieval rates are maintained. This is supported by Kuo et al. which reported 100% successful retrieval in a series of 50 patients where advanced manoeuvres were performed after standard methods had previously failed.[15] In a review of advanced techniques, Desai et al. illustrated that after 7 months of filter being in situ, the chances of the standard retrieval methods failing is high, with a calculated risk of 40.9% which continues to rise with increasing dwell time.[16] This evidence underscores the importance of both attempting advanced maneuvers when standard methods fail, as well as the necessity of reducing mean dwell time which can be done with regular follow up.

Although technically all IVC filters we insert are retrievable, 108 of the filters we inserted were made permanent (Table 1). When comparing to other studies this may seem high, for example Gregorio et al. reported 11.5% of IVC filters inserted were permanent; however, as the national neurosurgical center many of our patients unfortunately have a limited life expectancy. This reason dominates the reason for permanent insertion (49%). Being a neurosurgical center also results in a high number of trauma or neurooncological patients requiring prophylactic IVC filter insertion prior to major intracranial procedures in the setting of a current or previous VTE (34.1%). Permanent filters lead to complications such as filter migration or fracture, penetration outside of the vena cava wall and IVC stenosis or occlusion.[5] Further by improving retrieval practices and reviewing the indications for the conversion of retrievable filters to permanent, the institution can save on costs. Janne d’Othee et al. created a cost analysis model which demonstrated that retrievable versus permanent filter placement is financially beneficial even if only 41% of filters are removed.[17].

Limitations of this study include, the reliance on the IR fellow and IR administration staff to prospectively and correctly collate data in a timely manner. As fellows change every year, this is likely not optimal as there is loss of continuity. We think that the ideal person to monitor the database would be an IR practice nurse or physician associate but unfortunately these were not available to us.

We transitioned from the Celect filter to the ALN during the course of the study because of issues with filter leg penetration seen with the Celect filter.[18]

The prospective database also highlights the difficulty in assigning a status in terms of ‘intent to retrieve’, ‘permanent status’ or ‘unspecified’ to patients at the time of filter insertion. 47 patients who initially were in the unspecified group or intent to retrieve group were later converted to the ‘permanent’ group. This indicates that the database requires attention on a frequent basis and communication to referring physicians and patients must be ongoing.

## Conclusions

The removal of IVC filters, when indication for insertion has past, is no longer the sole responsibility of the referring physician but also the responsibility of the Interventionalist. Our retrieval rates of 92.5% of eligible IVC filters highlights the value of maintaining a prospective IVC filter registry with regular communication to referring physicians and patients.

## Data Availability

The datasets generated and/or analysed during the current study are not publicly available due patient confidentiality but are available from the corresponding author on reasonable request.

## Abbreviations

IVC: Inferior Vena Cava
GT: Gunther Tulip
VTE: Venous thromboembolism
DVT: Deep vein thrombosis
PE: Pulmonary embolism
FDA: Food and Drug Administration
CIRSE: Cardiovascular and Interventional Radiology Society of Europe
BSIR: British Society of Interventional Radiology
IR: Interventional Radiology
